# Synthesis and crystal structure of bis­(levofloxacindiium) di­aqua­tetra-μ-chlorido-penta­chlorido­tricopper(II) chloride

**DOI:** 10.1107/S2414314625009010

**Published:** 2025-10-24

**Authors:** Mahbuba Ishquvvatovna Bozorova, Khayit Khudainazarovich Turaev, Bekmurod Khurramovich Alimnazarov, Abdusamat Abdujabborovich Rasulov, Bakhtiyar Tulyaganovich Ibragimov, Jamshid Mengnorovich Ashurov

**Affiliations:** a Termez Branch of Tashkent State Medical University, 64 Islam Karimov Street, Termez City 132000, Uzbekistan; b Termez State University, Barkamol avlod street 43, Termez city, Uzbekistan; c Termez University of Economics and Service, 4-b Farovon Street, Termez 190111, Uzbekistan; d Institute of Bioorganic Chemistry, Academy of Sciences of Uzbekistan, 100125, M. Ulugbek Str 83, Tashkent, Uzbekistan; University of Antofagasta, Chile

**Keywords:** copper(II) complex, levofloxacindiium, crystal structure, hydrogen bonding.

## Abstract

The title compound, (C_18_H_22_FN_3_O_4_)_2_[Cu_3_Cl_9_(H_2_O)_2_]Cl, has been synthesized in the presence of levofloxacin, which acts as an organic component in the formation of the cationic species The asymmetric unit comprises one levofloxacin dication, one half of a trinuclear [Cu_3_Cl_9_(H_2_O)_2_]^3−^ anion and one half of an outer-sphere chloride anion. The central Cu^II^ atom, an apically coordinated chlorido ligand and the free chloride anion all reside on crystallographic twofold rotation axes, resulting in a unique symmetry-imposed configuration. Each Cu^II^ atom exhibits a five-coordinated distorted square-pyramidal coordination environment. The structure displays a pronounced Jahn–Teller distortion, evidenced by short basal Cu—Cl/O bonds ranging from 1.992 (4) to 2.3714 (12) Å and significantly elongated apical Cu—Cl inter­actions between 2.6547 (13) and 2.681 (2) Å. The crystal packing is stabilized by an extensive hydrogen-bonding network, with Cl and O atoms acting as acceptors for O—H⋯Cl, O—H⋯O, N—H⋯Cl and C—H⋯O inter­actions. These contacts link the cationic, anionic and neutral units into a robust tri-periodic supra­molecular architecture.

## Abstract 2

A trinuclear copper(II)–levofloxacin complex, (C_18_H_22_FN_3_O_4_)_2_[Cu_3_Cl_9_(H_2_O)_2_]Cl, was synthesized and its crystal structure determined. It features five-coordinated Cu^II^ centres with Jahn–Teller distortion and a three-dimensional hydrogen-bonded supra­molecular network.

## Graphical abstract

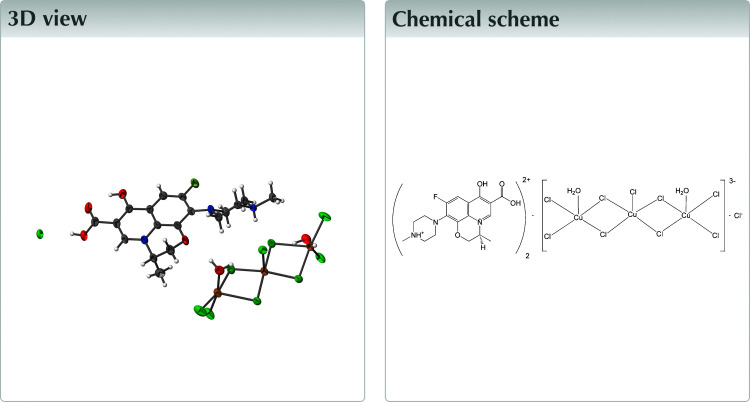

## Structure description

Levofloxacin, the optically pure *S*-(−)-enanti­omer of ofloxacin, is a prototypical third-generation fluoro­quinolone anti­biotic. Its mol­ecular structure, characterized by a fluorinated 4-quinolone core with carboxyl (C-3), keto (C-4) and piperazinyl (C-7) groups, confers a zwitterionic character at physiological pH. This specific configuration enhances its activity against Gram-positive and atypical pathogens while reducing central nervous system toxicity and drug–drug inter­actions (Scholar & Pratt, 2000 ▸; Owens & Ambrose, 2000 ▸; Podder *et al.*, 2024 ▸; Bano *et al.*, 2011 ▸). The structural profile is also responsible for its favourable pharmacokinetics, including high oral bioavailability (∼99%), minimal metabolism, predominant renal elimination and a notable ability to form stable metal complexes (DrugBank, 2023 ▸). The anti­biotic exerts its potent bactericidal effect through a balanced inhibition of bacterial DNA gyrase and topoisomerase IV. This action stabilizes the enzyme–DNA cleavage complex, ultimately disrupting DNA replication and segregation (Hooper, 2001 ▸). Beyond clinical pharmacology, levofloxacin displays remarkable versatility in the solid state. Neutral forms include the anhydrous API [Cambridge Structural Database (CSD; Groom *et al.*, 2016 ▸) refcodes LICWOM and LICWOM01; Freitas *et al.*, 2018 ▸] and hydrated forms such as the hemihydrate and monohydrate [YUJNUM and YUJPAU (Kitaoka *et al.*, 1995 ▸), YUJNUM01 (Gorman *et al.*, 2012 ▸), and YUJNUM02 and YUJPAU01 (Singh & Thakur, 2014 ▸)]. Monocationic salts (LevoH^+^) are exemplified by the 4-amino­salicylate sesquihydrate (AJOJOC; Ueda *et al.*, 2025 ▸); the di­hydrogen phosphate (CUKRIN), hydrogen phthalate (CUKROT), hydrogen sulfate (CUKVOX) and hydrogen citrate (CUKVUD) (Freitas *et al.*, 2025 ▸); the 2,6- and 3,5-di­hydroxy­benzoate salts (DOQQEJ and DOQQIN; Nugrahani *et al.*, 2023 ▸); the acesulfame salts (BUVKIQ and BUVKOW; Huang & Sun, 2025 ▸); and the fulfenamic acid salt (TURYEO; Nugrahani *et al.*, 2025 ▸). Dicationic salts LevoH_3_^2+^) or *levofloxacindiium* derivatives, are also numerous, including diperchlorate (GALFAD; Golovnev & Vasil’ev, 2016 ▸), tetra­bromido­cadmium(II) (GEKSAS; Vasiliev & Golovnev, 2011 ▸), tetra­kis­(levofloxacindiium) tris­[hexa­chlorido­stannate(IV)] (EPENAR; Golovnev *et al.*, 2021 ▸), and tetra­bromido­copper(II) (IJACII), tetra­chlorido­cobalt(II) (IJACOO) and tetra­bromido­zinc(II) (TUCJAF) (Vasiliev & Golovnev, 2019 ▸). These structures exhibit dense hydrogen-bonding and strong anion–cation inter­actions, stabilizing the dicationic species.

Levofloxacin also functions as a bidentate chelating ligand, coordinating through its carboxyl­ate and keto O atoms. Copper(II) complexes such as bis­(levofloxacin)–Cu^II^·MeOH·H_2_O (FUJJIF) and (2,2′-bi­pyridine)­chloro­levofloxacin–Cu^II^·H_2_O (FUJJOL) have been characterized (Galani *et al.*, 2014 ▸). Additional complexes include Zn^II^ (IGUCOE), Mg^II^ (PESWOA) and a wide variety of Cu^II^–phenanthroline and bi­pyridine adducts [SOWJUM and SOWKAT (Kumar *et al.*, 2019 ▸), TATKOS and TATKUY (Elhusseiny *et al.*, 2022 ▸), TAVDED (Bashir & Yousuf, 2022 ▸), VASCOL (Mubarak *et al.*, 2021 ▸) and WARXAP (Wang *et al.*, 2005 ▸)].

**Figure 1:**
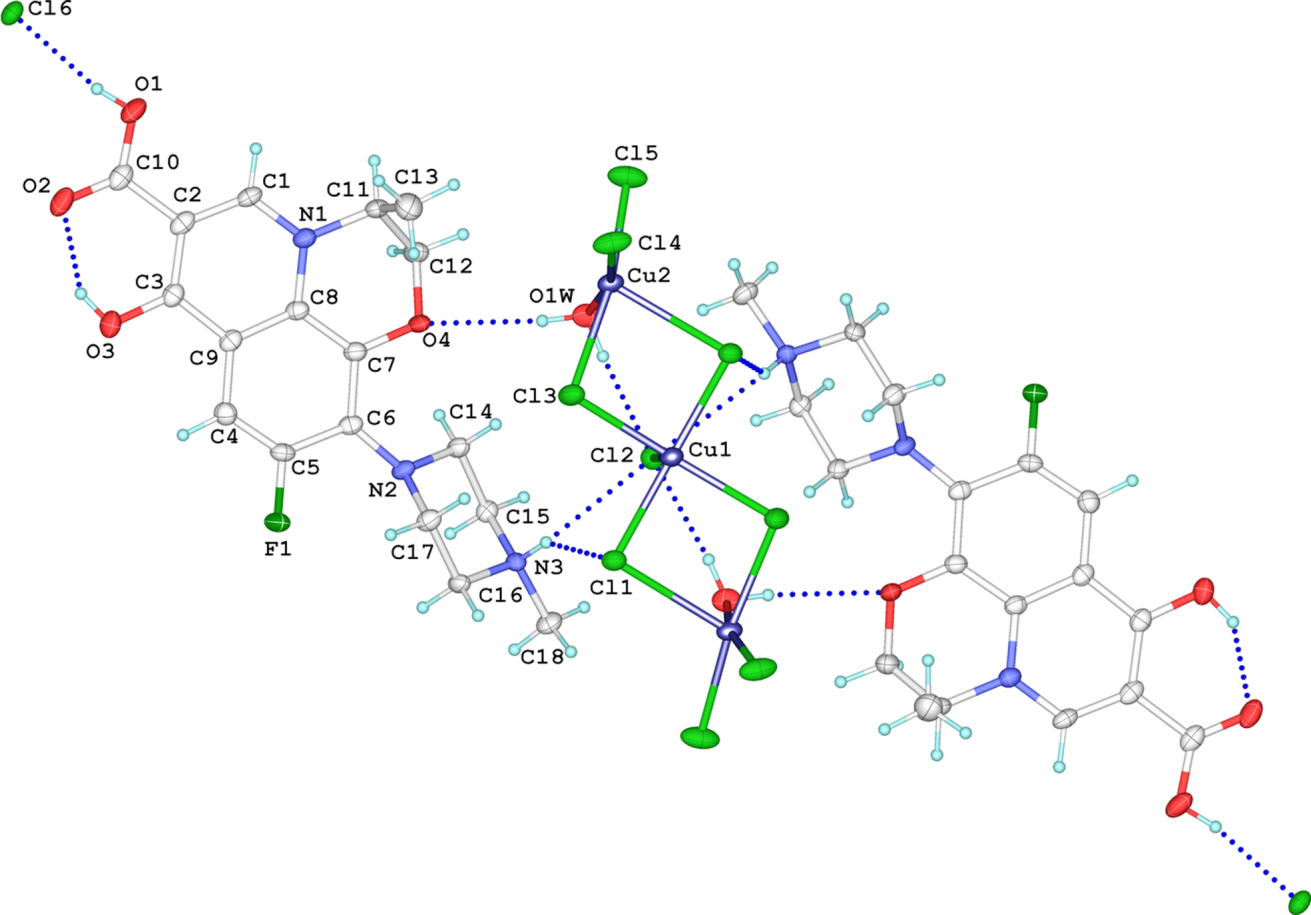
The mol­ecular structure of the title trinuclear copper(II)–levofloxacin complex, showing the atom-numbering scheme. Displacement ellipsoids are drawn at the 50% probability level. Atoms of the independent part are labelled; the remaining parts are generated by symmetry operation (−*x* + 1, *y*, −*z* + 1). Hydrogen bonds are shown as dashed lines.

In the present work, we report the synthesis and crystal structure of a new copper(II) coordination compound with levofloxacin, (C_18_H_22_FN_3_O_4_)_2_[Cu_3_Cl_9_(H_2_O)_2_]Cl, determined by single-crystal X-ray diffraction. The structure expands the family of levofloxacin-based coordination systems. In the complex, levofloxacin exists as a LevoH_3_^2+^ dication. One proton is located on the N3 atom of the piperazine ring, while the other is bonded to carbonyl atom O3. The C3—O3 bond length of 1.334 (6) Å clearly indicates protonation of the carbonyl group, being significantly longer than a typical C=O bond (∼1.26 Å). Comparable C—O bond elongations upon protonation have been reported for EPENAR (Golovnev *et al.*, 2021 ▸), GALFAD (Golovnev & Vasil’ev, 2016 ▸), and IJACII, IJACOO and TUCJAF (Vasiliev & Golovnev, 2019 ▸), where the values are around 1.34 (1) Å. The compound crystallizes as a trinuclear [Cu_3_Cl_9_(H_2_O)_2_]^3−^ anion paired with levofloxacindiium dications and an outer-sphere chloride anion. The asymmetric unit comprises one levofloxacindiium cation, one half of the [Cu_3_Cl_9_(H_2_O)_2_]^3−^ anion and one half of a chloride anion (Fig. 1 ▸). The central Cu^II^ atom (Cu1) lies on a crystallographic twofold axis shared with one apical chlorido ligand and the free chloride anion, resulting in a symmetry-imposed configuration of the anionic cluster.

The Cu1 atom is five-coordinated. According to the Addison descriptor [τ = (β − α)/60] (Addison *et al.*, 1984 ▸), the two largest angles are β = 176.85 (9)° (Cl1—Cu1—Cl1^i^) and α = 163.62 (10)° (Cl3—Cu1—Cl3^i^) [symmetry code: (i) −*x* + 1, *y*, −*z* + 1], giving τ = 0.22, indicative of a distinctly distorted square-pyramidal (SP) geometry. The coordination sphere consists of four chlorido ligands at the base of the pyramid [Cu1—Cl1 = 2.2830 (10) Å, Cu1—Cl3 = 2.3313 (12) Å], together with an elongated apical inter­action [Cu1—Cl2 = 2.681 (2) Å], consistent with a Jahn–Teller elongation. The polyhedral volume around Cu1 is 10.573 Å^3^, the largest among the three sites. The Berry pseudorotation coordinate is 84.7% along the *D*_3*h*_ → *C*_2*v*_ → *C*_4*v*_ path (Holmes 1984 ▸), *i.e.* close to the SP limit but more distorted than the terminal sites Cu2 sites. The latter are symmetry-equivalent within the trinuclear anion and crystallographically distinct from the central Cu1 atom. Each is five-coordinated and best described as SP, but with much less distortion. For Cu2, the largest angles are β = 167.76 (7)° (Cl3—Cu2—Cl5) and α = 167.10 (13)° (Cl4—Cu2—O1*W*), giving τ = 0.011, *i.e.* an almost ideal SP geometry (Addison *et al.*, 1984 ▸). The basal distances [Cu2—O1*W* = 1.992 (4) Å, Cu2—Cl4 = 2.2340 (17) Å, Cu2—Cl5 = 2.2664 (13) Å and Cu2—Cl3 = 2.3714 (12) Å] are comparable to those at Cu1, but the apical bond is less elongated [Cu2—Cl1 = 2.6547 (13) Å].

The polyhedral volume for Cu2 is 9.021 Å^3^, markedly smaller than that of Cu1, reflecting the reduced distortion. Both terminal sites are placed ≃ 89.9% along the Berry pseudorotation path to the SP end point (Holmes, 1984 ▸).

**Figure 2:**
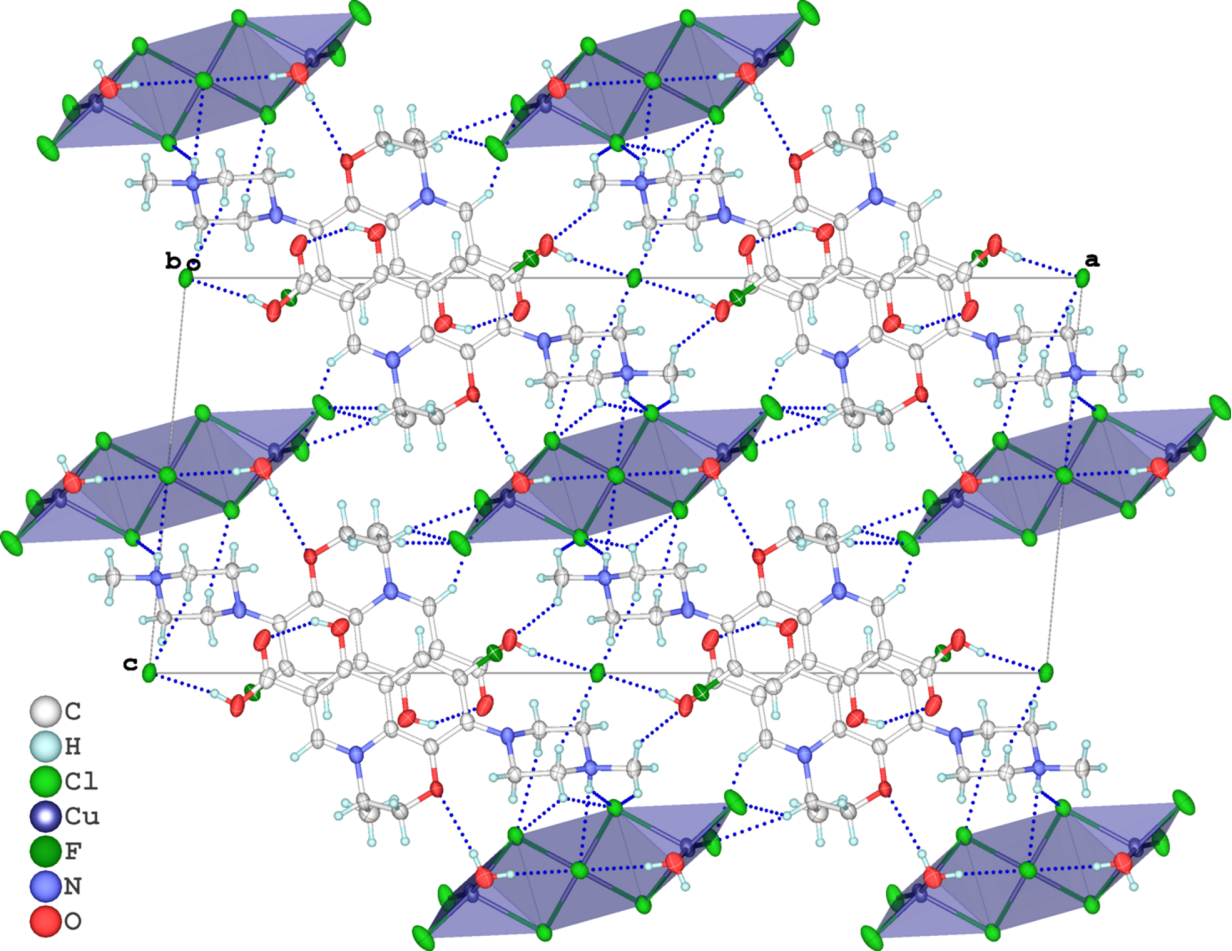
A packing diagram of the title complex, viewed along the [010] direction, showing the hydrogen-bonding network. Dashed lines indicate hydrogen bonds.

The crystal structure reveals an extensive hydrogen-bonding network of the O/N—H⋯Cl and O—H⋯O types, which connects the levofloxacin dications to the trinuclear [Cu_3_Cl_9_(H_2_O)_2_]^3−^ anion (Fig. 2 ▸ and Table 1 ▸). The crystal water mol­ecule O1*W* acts as a bifurcated hydrogen-bond donor: the O1*W*—H1*WA*⋯Cl2 inter­action [H⋯*A* = 2.25 Å, *D*⋯*A* = 3.097 (4) Å and *D*—H⋯*A* = 172°] is directed toward the apical chlorido ligand Cl2, while the O1*W*—H1*WB*⋯O4 contact [2.38 Å, 3.214 (5) Å and 167°] is established with the oxazinic ring O atom (O4) of the levofloxacin moiety. The carb­oxyl proton O1—H1 forms a strong hydrogen bond with the terminal (outer-sphere) chloride Cl6 [2.19 Å, 2.995 (4) Å and 169°], thus explicitly connecting the –COOH group to a free chloride anion. Notably, the piperazinyl proton N3—H3*A* engages in a bifurcated hydrogen bond with both Cl1 and Cl2 [2.57 (5)/2.69 (5) Å, 3.234 (5)/3.292 (4) Å and 139 (4)/132 (5)°], which effectively inter­connects adjacent anionic clusters. An intra­molecular O3—H3⋯O2 hydrogen bond [1.79 Å, 2.522 (5) Å and 147°] stabilizes the conformation of the levofloxacin backbone. Additionally, a weak inter­molecular contact C17—H17*A*⋯O2^i^ [2.72 Å, 3.294 (7) Å and 119°; symmetry code: (i) −*x* + 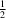, *y* − 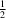, −*z*] further assists the crystal packing. Only three chlorido ligands – Cl1, Cl2 and Cl6 – serve as hydrogen-bond acceptors, while other outer-sphere chlorides are not involved. The combined hydrogen-bond pattern comprising O1*W*—H1*WA*⋯Cl2 and O1—H1⋯Cl6 propagates into chains along the [101] direction. These chains are inter­connected by N3—H3*A*⋯Cl1/Cl2 bridges to generate layers, which are further linked into a robust tri-periodic supra­molecular framework *via* O1*W*—H1*WB*⋯O4 and C—H⋯O inter­actions. Notably, a discrete 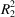
(14) graph-set motif is formed by the complementary O1*W*—H1*WB*⋯O4 and N3—H3*A*⋯Cl1 hydrogen bonds, further reinforcing the structural cohesion.

## Synthesis and crystallization

All reagents and solvents were of analytical grade and were used as received without further purification. Levofloxacin hemihydrate (commercial sample, C_18_H_20_FN_3_O_4_·0.5H_2_O, 1 mmol, 0.37 g) was dissolved in a mixed solvent of distilled water (5 ml) and ethanol (10 ml) with the addition of a few drops of concentrated hydro­chloric acid to facilitate dissolution. A solution of copper(II) chloride dihydrate (CuCl_2_·2H_2_O, 1.5 mmol, 0.26 g) in distilled water (5 ml) was added dropwise under constant stirring. The resulting clear-blue solution was left to evaporate slowly at room temperature. After about 5 d, light-blue crystals suitable for single-crystal X-ray diffraction were obtained and collected by filtration.

## Refinement

Crystal data, data collection, and structure refinement details are summarized in Table 2 ▸.

## Supplementary Material

Crystal structure: contains datablock(s) I. DOI: 10.1107/S2414314625009010/bx4037sup1.cif

Structure factors: contains datablock(s) I. DOI: 10.1107/S2414314625009010/bx4037Isup2.hkl

CCDC reference: 2495754

Additional supporting information:  crystallographic information; 3D view; checkCIF report

Additional supporting information:  crystallographic information; 3D view; checkCIF report

## Figures and Tables

**Table 1:** Hydrogen-bond geometry (Å, °)

*D*—H⋯*A*	*D*—H	H⋯*A*	*D*⋯*A*	*D*—H⋯*A*
O1*W*—H1*WA*⋯Cl2	0.85	2.25	3.097 (4)	172
O1*W*—H1*WB*⋯O4	0.85	2.38	3.214 (5)	167
O1—H1⋯Cl6	0.82	2.19	2.995 (4)	169
O3—H3⋯O2	0.82	1.79	2.522 (5)	147
N3—H3*A*⋯Cl1	0.82 (5)	2.57 (5)	3.234 (5)	139 (4)
N3—H3*A*⋯Cl2	0.82 (5)	2.69 (5)	3.292 (4)	132 (5)
C17—H17*A*⋯O2^i^	0.97	2.72	3.294 (7)	119

Symmetry code: (i) 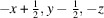.

**Table 2:** Experimental details

Crystal data	
Chemical formula	(C_18_H_22_FN_3_O_4_)_2_[Cu_3_Cl_9_(H_2_O)_2_]Cl
*M*_r_	1307.92
Crystal system, space group	Monoclinic, *C*2
Temperature (K)	291
*a*, *b*, *c* (Å)	28.5670 (4), 6.8201 (1), 12.6444 (2)
β (°)	95.278 (1)
*V* (Å^3^)	2453.06 (6)
*Z*	2
Radiation type	Cu *K*α
μ (mm^−1^)	7.11
Crystal size (mm)	0.25 × 0.2 × 0.16

Data collection	
Diffractometer	Rigaku XtaLAB Synergy Single source diffractometer with a HyPix3000 detector
Absorption correction	Multi-scan (*CrysAlis PRO*; Rigaku OD, 2022 ▸)
*T*_min_, *T*_max_	0.125, 1.000
No. of measured, independent and observed [*I* > 2σ(*I*)] reflections	6918, 3765, 3542
*R*_int_	0.036
(sin θ/λ)_max_ (Å^−1^)	0.615

Refinement	
*R*[*F*^2^ > 2σ(*F*^2^)], *wR*(*F*^2^), *S*	0.036, 0.090, 1.02
No. of reflections	3765
No. of parameters	314
No. of restraints	1
H-atom treatment	H atoms treated by a mixture of independent and constrained refinement
Δρ_max_, Δρ_min_ (e Å^−3^)	0.36, −0.46
Absolute structure	Flack *x* determined using 1042 quotients [(*I*^+^)−(*I*^−^)]/[(*I*^+^)+(*I*^−^)] (Parsons *et al.*, 2013 ▸)
Absolute structure parameter	0.037 (14)

Computer programs: *CrysAlis PRO* (Rigaku OD, 2022 ▸), *SHELXT2018* (Sheldrick, 2015*a* ▸), *SHELXL2019* (Sheldrick, 2015*b* ▸), *OLEX2* (Dolomanov *et al.*, 2009 ▸) and *publCIF* (Westrip, 2010 ▸).

## full crystallographic data

### 11-Carboxy-7-fluoro-10-hydroxy-2-methyl-6-(4-methylpiperazin-4-ium-1-yl)-4-oxa-1λ5-azatricyclo[7.3.1.05,13]trideca-1(12),5,7,9(13),10-pentaen-1-ylium [aquadichloridocopper(II)]-di-µ-chlorido-[chloridocopper(II)]-di-µ-chlorido-[aquadichloridocopper(II)] chloride Crystal data

**Table:**

(C_18_H_22_FN_3_O_4_)_2_[Cu_3_Cl_9_(H_2_O)_2_]Cl	*F*(000) = 1322
*M**_r_* = 1307.92	*D*_x_ = 1.771 Mg m^−^^3^
Monoclinic, *C*2	Cu *K*α radiation, λ = 1.54184 Å
*a* = 28.5670 (4) Å	Cell parameters from 4945 reflections
*b* = 6.8201 (1) Å	θ = 3.1–70.7°
*c* = 12.6444 (2) Å	µ = 7.11 mm^−^^1^
β = 95.278 (1)°	*T* = 291 K
*V* = 2453.06 (6) Å^3^	Block, light blue
*Z* = 2	0.25 × 0.2 × 0.16 mm

### 11-Carboxy-7-fluoro-10-hydroxy-2-methyl-6-(4-methylpiperazin-4-ium-1-yl)-4-oxa-1λ5-azatricyclo[7.3.1.05,13]trideca-1(12),5,7,9(13),10-pentaen-1-ylium [aquadichloridocopper(II)]-di-µ-chlorido-[chloridocopper(II)]-di-µ-chlorido-[aquadichloridocopper(II)] chloride Data collection

**Table:**

Rigaku XtaLAB Synergy Single source diffractometer with a HyPix3000 detector	3765 independent reflections
Radiation source: micro-focus sealed X-ray tube, PhotonJet (Cu) X-ray Source	3542 reflections with *I* > 2σ(*I*)
Mirror monochromator	*R*_int_ = 0.036
Detector resolution: 10.0000 pixels mm^-1^	θ_max_ = 71.5°, θ_min_ = 3.1°
ω scans	*h* = −31→34
Absorption correction: multi-scan (CrysAlis PRO; Rigaku OD, 2022)	*k* = −7→8
*T*_min_ = 0.125, *T*_max_ = 1.000	*l* = −15→15
6918 measured reflections	

### 11-Carboxy-7-fluoro-10-hydroxy-2-methyl-6-(4-methylpiperazin-4-ium-1-yl)-4-oxa-1λ5-azatricyclo[7.3.1.05,13]trideca-1(12),5,7,9(13),10-pentaen-1-ylium [aquadichloridocopper(II)]-di-µ-chlorido-[chloridocopper(II)]-di-µ-chlorido-[aquadichloridocopper(II)] chloride Refinement

**Table:**

Refinement on *F*^2^	H atoms treated by a mixture of independent and constrained refinement
Least-squares matrix: full	*w* = 1/[σ^2^(*F*_o_^2^) + (0.0452*P*)^2^] where *P* = (*F*_o_^2^ + 2*F*_c_^2^)/3
*R*[*F*^2^ > 2σ(*F*^2^)] = 0.036	(Δ/σ)_max_ < 0.001
*wR*(*F*^2^) = 0.090	Δρ_max_ = 0.36 e Å^−^^3^
*S* = 1.02	Δρ_min_ = −0.46 e Å^−^^3^
3765 reflections	Extinction correction: SHELXL2019 (Sheldrick, 2015b), Fc^*^=kFc[1+0.001xFc^2^λ^3^/sin(2θ)]^-1/4^
314 parameters	Extinction coefficient: 0.00032 (6)
1 restraint	Absolute structure: Flack *x* determined using 1042 quotients [(I+)-(I-)]/[(I+)+(I-)] (Parsons *et al.*, 2013)
Hydrogen site location: mixed	Absolute structure parameter: 0.037 (14)

### 11-Carboxy-7-fluoro-10-hydroxy-2-methyl-6-(4-methylpiperazin-4-ium-1-yl)-4-oxa-1λ5-azatricyclo[7.3.1.05,13]trideca-1(12),5,7,9(13),10-pentaen-1-ylium [aquadichloridocopper(II)]-di-µ-chlorido-[chloridocopper(II)]-di-µ-chlorido-[aquadichloridocopper(II)] chloride Special details

**Table:**

Geometry. All esds (except the esd in the dihedral angle between two l.s. planes) are estimated using the full covariance matrix. The cell esds are taken into account individually in the estimation of esds in distances, angles and torsion angles; correlations between esds in cell parameters are only used when they are defined by crystal symmetry. An approximate (isotropic) treatment of cell esds is used for estimating esds involving l.s. planes.
Refinement. All non-H atoms were refined with anisotropic displacement parameters. H atoms were placed in calculated positions and refined using a riding model, with C—H distances of 0.93 (aromatic), 0.96 (methyl) and 0.97 Å (methylene), and with O—H = 0.82 Å. The isotropic displacement parameters were set to *U*_iso_(H) = 1.5*U*_eq_(C) for methyl groups and 1.2*U*_eq_(*X*) for other H atoms (*X* = C, O). The H3*A* atom, attached to the N3 atom of the piperazine ring, was located from a difference Fourier map and refined freely.

### 11-Carboxy-7-fluoro-10-hydroxy-2-methyl-6-(4-methylpiperazin-4-ium-1-yl)-4-oxa-1λ5-azatricyclo[7.3.1.05,13]trideca-1(12),5,7,9(13),10-pentaen-1-ylium [aquadichloridocopper(II)]-di-µ-chlorido-[chloridocopper(II)]-di-µ-chlorido-[aquadichloridocopper(II)] chloride Fractional atomic coordinates and isotropic or equivalent isotropic displacement parameters (Å^2^)

**Table:**

	*x*	*y*	*z*	*U*_iso_*/*U*_eq_	
Cu1	0.500000	0.35086 (18)	0.500000	0.0341 (3)	
Cu2	0.38177 (2)	0.38447 (12)	0.55789 (5)	0.0340 (2)	
Cl1	0.53295 (4)	0.3416 (2)	0.34229 (9)	0.0344 (3)	
Cl2	0.500000	0.7439 (3)	0.500000	0.0357 (4)	
Cl3	0.42531 (4)	0.3022 (2)	0.41328 (9)	0.0410 (3)	
Cl4	0.35486 (5)	0.0787 (2)	0.57104 (14)	0.0533 (4)	
Cl5	0.33305 (6)	0.5117 (3)	0.67187 (13)	0.0575 (4)	
O1W	0.39453 (13)	0.6613 (6)	0.5191 (3)	0.0421 (9)	
H1WA	0.423490	0.673977	0.510036	0.063*	
H1WB	0.380251	0.686336	0.458677	0.063*	
Cl6	0.000000	0.6707 (3)	0.000000	0.0388 (4)	
F1	0.38299 (10)	0.6658 (6)	−0.0497 (2)	0.0469 (9)	
O1	0.10162 (12)	0.6630 (8)	0.0830 (3)	0.0528 (12)	
H1	0.075099	0.663550	0.052107	0.079*	
O2	0.12245 (12)	0.6620 (8)	−0.0827 (3)	0.0487 (10)	
O3	0.20818 (12)	0.6512 (8)	−0.1154 (3)	0.0449 (10)	
H3	0.179639	0.649827	−0.130435	0.067*	
O4	0.33240 (11)	0.6878 (7)	0.2944 (3)	0.0440 (11)	
N1	0.23920 (13)	0.6454 (7)	0.2074 (3)	0.0331 (9)	
N2	0.40597 (12)	0.7098 (7)	0.1604 (3)	0.0315 (10)	
N3	0.50208 (13)	0.7622 (7)	0.2403 (3)	0.0277 (8)	
H3A	0.5021 (16)	0.686 (8)	0.290 (4)	0.021 (13)*	
C1	0.19449 (16)	0.6429 (9)	0.1670 (4)	0.0356 (12)	
H1A	0.170882	0.636910	0.212822	0.043*	
C2	0.18220 (16)	0.6491 (9)	0.0587 (4)	0.0349 (11)	
C3	0.21748 (16)	0.6492 (9)	−0.0101 (4)	0.0321 (11)	
C4	0.30286 (17)	0.6466 (9)	−0.0342 (4)	0.0346 (11)	
H4	0.297169	0.635905	−0.107555	0.042*	
C5	0.34713 (16)	0.6590 (9)	0.0117 (4)	0.0333 (11)	
C6	0.35981 (16)	0.6802 (8)	0.1243 (4)	0.0295 (10)	
C7	0.32186 (16)	0.6774 (8)	0.1874 (4)	0.0310 (11)	
C8	0.27564 (16)	0.6579 (8)	0.1421 (4)	0.0303 (10)	
C9	0.26541 (16)	0.6501 (8)	0.0300 (4)	0.0300 (10)	
C10	0.13251 (17)	0.6568 (9)	0.0137 (4)	0.0386 (12)	
C11	0.25185 (17)	0.6273 (10)	0.3248 (4)	0.0389 (13)	
H11	0.225705	0.677275	0.361990	0.047*	
C12	0.29370 (16)	0.7558 (11)	0.3505 (4)	0.0444 (15)	
H12A	0.285978	0.889830	0.329984	0.053*	
H12B	0.302612	0.753430	0.426351	0.053*	
C13	0.2597 (2)	0.4147 (12)	0.3537 (5)	0.0571 (18)	
H13A	0.232286	0.340147	0.329661	0.086*	
H13B	0.265588	0.402621	0.429443	0.086*	
H13C	0.286299	0.365943	0.320569	0.086*	
C14	0.41798 (15)	0.8238 (9)	0.2581 (4)	0.0331 (11)	
H14A	0.418628	0.738332	0.319502	0.040*	
H14B	0.394469	0.924412	0.265085	0.040*	
C15	0.46582 (15)	0.9176 (8)	0.2524 (4)	0.0297 (10)	
H15A	0.464720	1.006792	0.192457	0.036*	
H15B	0.474226	0.992360	0.316596	0.036*	
C16	0.48900 (16)	0.6479 (9)	0.1409 (4)	0.0337 (12)	
H16A	0.512102	0.545775	0.133594	0.040*	
H16B	0.488915	0.734107	0.079898	0.040*	
C17	0.44080 (16)	0.5565 (9)	0.1442 (4)	0.0352 (11)	
H17A	0.432042	0.488268	0.078018	0.042*	
H17B	0.441524	0.461910	0.201588	0.042*	
C18	0.55014 (16)	0.8450 (10)	0.2418 (4)	0.0415 (13)	
H18A	0.572518	0.740180	0.240626	0.062*	
H18B	0.556929	0.921360	0.305146	0.062*	
H18C	0.552073	0.926993	0.180675	0.062*	

### 11-Carboxy-7-fluoro-10-hydroxy-2-methyl-6-(4-methylpiperazin-4-ium-1-yl)-4-oxa-1λ5-azatricyclo[7.3.1.05,13]trideca-1(12),5,7,9(13),10-pentaen-1-ylium [aquadichloridocopper(II)]-di-µ-chlorido-[chloridocopper(II)]-di-µ-chlorido-[aquadichloridocopper(II)] chloride Atomic displacement parameters (Å^2^)

**Table:**

	*U*^11^	*U*^22^	*U*^33^	*U*^12^	*U*^13^	*U*^23^
Cu1	0.0265 (5)	0.0428 (7)	0.0343 (5)	0.000	0.0093 (4)	0.000
Cu2	0.0270 (3)	0.0348 (5)	0.0414 (4)	0.0016 (3)	0.0104 (3)	−0.0030 (3)
Cl1	0.0302 (5)	0.0385 (8)	0.0355 (5)	−0.0010 (5)	0.0084 (4)	−0.0042 (5)
Cl2	0.0363 (8)	0.0391 (10)	0.0325 (7)	0.000	0.0070 (6)	0.000
Cl3	0.0309 (6)	0.0535 (9)	0.0395 (6)	−0.0015 (6)	0.0085 (4)	−0.0123 (6)
Cl4	0.0400 (7)	0.0355 (8)	0.0886 (11)	−0.0020 (6)	0.0279 (7)	−0.0080 (7)
Cl5	0.0624 (9)	0.0449 (9)	0.0715 (9)	0.0008 (8)	0.0401 (8)	−0.0073 (8)
O1W	0.0398 (19)	0.040 (2)	0.048 (2)	0.0040 (19)	0.0110 (16)	0.0048 (19)
Cl6	0.0224 (7)	0.0507 (12)	0.0428 (9)	0.000	−0.0003 (6)	0.000
F1	0.0310 (15)	0.077 (3)	0.0341 (14)	−0.0056 (17)	0.0111 (12)	−0.0051 (16)
O1	0.0233 (17)	0.077 (4)	0.057 (2)	−0.004 (2)	−0.0012 (16)	0.011 (2)
O2	0.0305 (18)	0.060 (3)	0.053 (2)	0.001 (2)	−0.0100 (16)	−0.002 (2)
O3	0.0355 (18)	0.060 (3)	0.0369 (18)	−0.002 (2)	−0.0067 (15)	0.0006 (19)
O4	0.0203 (15)	0.082 (3)	0.0300 (16)	−0.0046 (19)	0.0054 (12)	−0.0035 (19)
N1	0.0248 (19)	0.040 (3)	0.035 (2)	−0.0012 (19)	0.0050 (16)	−0.002 (2)
N2	0.0210 (18)	0.039 (3)	0.034 (2)	−0.0016 (18)	0.0037 (15)	−0.0100 (19)
N3	0.0231 (18)	0.034 (2)	0.0259 (18)	−0.0039 (17)	0.0044 (14)	0.0013 (18)
C1	0.021 (2)	0.040 (3)	0.046 (3)	−0.001 (2)	0.0055 (19)	−0.001 (3)
C2	0.026 (2)	0.028 (3)	0.050 (3)	−0.002 (2)	0.000 (2)	−0.002 (2)
C3	0.029 (2)	0.028 (3)	0.038 (2)	−0.001 (2)	−0.0012 (19)	0.000 (2)
C4	0.035 (3)	0.036 (3)	0.032 (2)	−0.002 (2)	0.0031 (19)	−0.004 (2)
C5	0.030 (2)	0.038 (3)	0.034 (2)	−0.001 (2)	0.0122 (19)	0.001 (2)
C6	0.026 (2)	0.026 (3)	0.038 (2)	−0.003 (2)	0.0066 (18)	−0.002 (2)
C7	0.027 (2)	0.035 (3)	0.033 (2)	−0.001 (2)	0.0071 (18)	−0.002 (2)
C8	0.025 (2)	0.031 (3)	0.035 (2)	−0.004 (2)	0.0060 (18)	−0.003 (2)
C9	0.026 (2)	0.025 (3)	0.038 (2)	−0.002 (2)	0.0009 (18)	−0.002 (2)
C10	0.030 (2)	0.032 (3)	0.052 (3)	−0.002 (2)	−0.004 (2)	0.002 (3)
C11	0.024 (2)	0.058 (4)	0.036 (3)	−0.001 (2)	0.0081 (19)	0.000 (3)
C12	0.032 (2)	0.067 (4)	0.035 (3)	−0.004 (3)	0.008 (2)	−0.010 (3)
C13	0.050 (3)	0.073 (5)	0.048 (3)	−0.006 (4)	0.006 (3)	0.011 (3)
C14	0.026 (2)	0.042 (3)	0.032 (2)	0.000 (2)	0.0042 (17)	−0.009 (2)
C15	0.029 (2)	0.027 (3)	0.033 (2)	−0.001 (2)	0.0018 (17)	0.000 (2)
C16	0.026 (2)	0.049 (3)	0.027 (2)	−0.001 (2)	0.0057 (17)	−0.009 (2)
C17	0.028 (2)	0.037 (3)	0.041 (3)	−0.001 (2)	0.0037 (19)	−0.008 (2)
C18	0.025 (2)	0.052 (4)	0.047 (3)	−0.007 (3)	0.0035 (19)	0.002 (3)

### 11-Carboxy-7-fluoro-10-hydroxy-2-methyl-6-(4-methylpiperazin-4-ium-1-yl)-4-oxa-1λ5-azatricyclo[7.3.1.05,13]trideca-1(12),5,7,9(13),10-pentaen-1-ylium [aquadichloridocopper(II)]-di-µ-chlorido-[chloridocopper(II)]-di-µ-chlorido-[aquadichloridocopper(II)] chloride Geometric parameters (Å, º)

**Table:**

Cu1—Cl1^i^	2.2830 (10)	C2—C3	1.390 (7)
Cu1—Cl1	2.2830 (10)	C2—C10	1.481 (7)
Cu1—Cl2	2.681 (2)	C3—C9	1.416 (6)
Cu1—Cl3^i^	2.3313 (12)	C4—H4	0.9300
Cu1—Cl3	2.3313 (12)	C4—C5	1.345 (7)
Cu2—Cl1^i^	2.6547 (13)	C4—C9	1.401 (7)
Cu2—Cl3	2.3714 (12)	C5—C6	1.444 (7)
Cu2—Cl4	2.2340 (17)	C6—C7	1.404 (6)
Cu2—Cl5	2.2664 (13)	C7—C8	1.397 (6)
Cu2—O1W	1.992 (4)	C8—C9	1.421 (6)
O1W—H1WA	0.8501	C11—H11	0.9800
O1W—H1WB	0.8501	C11—C12	1.494 (8)
F1—C5	1.342 (5)	C11—C13	1.507 (10)
O1—H1	0.8200	C12—H12A	0.9700
O1—C10	1.300 (7)	C12—H12B	0.9700
O2—C10	1.227 (6)	C13—H13A	0.9600
O3—H3	0.8200	C13—H13B	0.9600
O3—C3	1.334 (6)	C13—H13C	0.9600
O4—C7	1.360 (6)	C14—H14A	0.9700
O4—C12	1.444 (6)	C14—H14B	0.9700
N1—C1	1.331 (6)	C14—C15	1.517 (6)
N1—C8	1.390 (6)	C15—H15A	0.9700
N1—C11	1.500 (6)	C15—H15B	0.9700
N2—C6	1.370 (6)	C16—H16A	0.9700
N2—C14	1.473 (6)	C16—H16B	0.9700
N2—C17	1.470 (7)	C16—C17	1.515 (7)
N3—H3A	0.82 (5)	C17—H17A	0.9700
N3—C15	1.500 (6)	C17—H17B	0.9700
N3—C16	1.496 (6)	C18—H18A	0.9600
N3—C18	1.483 (6)	C18—H18B	0.9600
C1—H1A	0.9300	C18—H18C	0.9600
C1—C2	1.382 (7)		
			
Cl1^i^—Cu1—Cl1	176.85 (9)	O4—C7—C8	121.9 (4)
Cl1—Cu1—Cl2	91.58 (4)	C8—C7—C6	121.2 (4)
Cl1^i^—Cu1—Cl2	91.58 (4)	N1—C8—C7	119.6 (4)
Cl1^i^—Cu1—Cl3	88.50 (4)	N1—C8—C9	119.6 (4)
Cl1^i^—Cu1—Cl3^i^	91.05 (4)	C7—C8—C9	120.9 (4)
Cl1—Cu1—Cl3^i^	88.50 (4)	C3—C9—C8	117.4 (4)
Cl1—Cu1—Cl3	91.05 (4)	C4—C9—C3	123.9 (5)
Cl3^i^—Cu1—Cl2	98.19 (5)	C4—C9—C8	118.7 (4)
Cl3—Cu1—Cl2	98.19 (5)	O1—C10—C2	115.4 (5)
Cl3^i^—Cu1—Cl3	163.62 (10)	O2—C10—O1	123.9 (5)
Cl3—Cu2—Cl1^i^	79.46 (4)	O2—C10—C2	120.7 (5)
Cl4—Cu2—Cl1^i^	99.72 (6)	N1—C11—H11	108.7
Cl4—Cu2—Cl3	92.57 (6)	N1—C11—C13	109.7 (5)
Cl4—Cu2—Cl5	94.27 (6)	C12—C11—N1	106.4 (4)
Cl5—Cu2—Cl1^i^	109.28 (6)	C12—C11—H11	108.7
Cl5—Cu2—Cl3	167.76 (7)	C12—C11—C13	114.4 (5)
O1W—Cu2—Cl1^i^	92.32 (12)	C13—C11—H11	108.7
O1W—Cu2—Cl3	85.06 (11)	O4—C12—C11	109.8 (5)
O1W—Cu2—Cl4	167.10 (13)	O4—C12—H12A	109.7
O1W—Cu2—Cl5	86.02 (11)	O4—C12—H12B	109.7
Cu1—Cl1—Cu2^i^	90.90 (4)	C11—C12—H12A	109.7
Cu1—Cl3—Cu2	97.23 (5)	C11—C12—H12B	109.7
Cu2—O1W—H1WA	109.4	H12A—C12—H12B	108.2
Cu2—O1W—H1WB	109.2	C11—C13—H13A	109.5
H1WA—O1W—H1WB	104.5	C11—C13—H13B	109.5
C10—O1—H1	109.5	C11—C13—H13C	109.5
C3—O3—H3	109.5	H13A—C13—H13B	109.5
C7—O4—C12	113.3 (4)	H13A—C13—H13C	109.5
C1—N1—C8	121.1 (4)	H13B—C13—H13C	109.5
C1—N1—C11	120.9 (4)	N2—C14—H14A	109.9
C8—N1—C11	117.9 (4)	N2—C14—H14B	109.9
C6—N2—C14	120.0 (4)	N2—C14—C15	108.9 (3)
C6—N2—C17	119.5 (4)	H14A—C14—H14B	108.3
C17—N2—C14	112.5 (4)	C15—C14—H14A	109.9
C15—N3—H3A	109 (3)	C15—C14—H14B	109.9
C16—N3—H3A	107 (4)	N3—C15—C14	109.9 (4)
C16—N3—C15	109.4 (4)	N3—C15—H15A	109.7
C18—N3—H3A	107 (3)	N3—C15—H15B	109.7
C18—N3—C15	112.1 (4)	C14—C15—H15A	109.7
C18—N3—C16	111.6 (4)	C14—C15—H15B	109.7
N1—C1—H1A	119.1	H15A—C15—H15B	108.2
N1—C1—C2	121.8 (4)	N3—C16—H16A	109.6
C2—C1—H1A	119.1	N3—C16—H16B	109.6
C1—C2—C3	119.1 (4)	N3—C16—C17	110.4 (3)
C1—C2—C10	121.9 (5)	H16A—C16—H16B	108.1
C3—C2—C10	119.0 (5)	C17—C16—H16A	109.6
O3—C3—C2	122.4 (4)	C17—C16—H16B	109.6
O3—C3—C9	117.1 (4)	N2—C17—C16	109.8 (5)
C2—C3—C9	120.6 (5)	N2—C17—H17A	109.7
C5—C4—H4	120.4	N2—C17—H17B	109.7
C5—C4—C9	119.1 (4)	C16—C17—H17A	109.7
C9—C4—H4	120.4	C16—C17—H17B	109.7
F1—C5—C4	119.3 (4)	H17A—C17—H17B	108.2
F1—C5—C6	115.6 (4)	N3—C18—H18A	109.5
C4—C5—C6	124.9 (4)	N3—C18—H18B	109.5
N2—C6—C5	119.3 (4)	N3—C18—H18C	109.5
N2—C6—C7	125.7 (4)	H18A—C18—H18B	109.5
C7—C6—C5	115.0 (4)	H18A—C18—H18C	109.5
O4—C7—C6	116.9 (4)	H18B—C18—H18C	109.5
			
F1—C5—C6—N2	1.6 (8)	C5—C6—C7—C8	−0.8 (8)
F1—C5—C6—C7	178.6 (5)	C6—N2—C14—C15	−152.7 (5)
O3—C3—C9—C4	−2.1 (9)	C6—N2—C17—C16	153.7 (4)
O3—C3—C9—C8	176.9 (5)	C6—C7—C8—N1	176.6 (5)
O4—C7—C8—N1	−1.1 (9)	C6—C7—C8—C9	−3.2 (9)
O4—C7—C8—C9	179.1 (5)	C7—O4—C12—C11	56.3 (7)
N1—C1—C2—C3	2.8 (10)	C7—C8—C9—C3	−174.1 (5)
N1—C1—C2—C10	−176.8 (6)	C7—C8—C9—C4	5.0 (8)
N1—C8—C9—C3	6.1 (8)	C8—N1—C1—C2	1.2 (9)
N1—C8—C9—C4	−174.8 (5)	C8—N1—C11—C12	38.1 (7)
N1—C11—C12—O4	−61.6 (6)	C8—N1—C11—C13	−86.1 (6)
N2—C6—C7—O4	−6.2 (9)	C9—C4—C5—F1	−176.6 (5)
N2—C6—C7—C8	176.0 (5)	C9—C4—C5—C6	−1.4 (9)
N2—C14—C15—N3	−58.9 (5)	C10—C2—C3—O3	−1.7 (9)
N3—C16—C17—N2	56.6 (6)	C10—C2—C3—C9	177.4 (5)
C1—N1—C8—C7	174.4 (5)	C11—N1—C1—C2	−176.6 (5)
C1—N1—C8—C9	−5.8 (9)	C11—N1—C8—C7	−7.8 (8)
C1—N1—C11—C12	−144.0 (6)	C11—N1—C8—C9	172.0 (5)
C1—N1—C11—C13	91.7 (6)	C12—O4—C7—C6	158.5 (5)
C1—C2—C3—O3	178.7 (5)	C12—O4—C7—C8	−23.7 (8)
C1—C2—C3—C9	−2.2 (9)	C13—C11—C12—O4	59.6 (6)
C1—C2—C10—O1	1.9 (9)	C14—N2—C6—C5	149.8 (5)
C1—C2—C10—O2	−179.9 (6)	C14—N2—C6—C7	−26.8 (9)
C2—C3—C9—C4	178.8 (6)	C14—N2—C17—C16	−57.5 (5)
C2—C3—C9—C8	−2.2 (8)	C15—N3—C16—C17	−58.2 (6)
C3—C2—C10—O1	−177.7 (6)	C16—N3—C15—C14	59.5 (5)
C3—C2—C10—O2	0.5 (9)	C17—N2—C6—C5	−63.6 (7)
C4—C5—C6—N2	−173.9 (6)	C17—N2—C6—C7	119.7 (6)
C4—C5—C6—C7	3.1 (9)	C17—N2—C14—C15	58.6 (6)
C5—C4—C9—C3	176.3 (5)	C18—N3—C15—C14	−176.1 (4)
C5—C4—C9—C8	−2.8 (9)	C18—N3—C16—C17	177.1 (5)
C5—C6—C7—O4	177.0 (5)		

Symmetry code: (i) −*x*+1, *y*, −*z*+1.

### 11-Carboxy-7-fluoro-10-hydroxy-2-methyl-6-(4-methylpiperazin-4-ium-1-yl)-4-oxa-1λ5-azatricyclo[7.3.1.05,13]trideca-1(12),5,7,9(13),10-pentaen-1-ylium [aquadichloridocopper(II)]-di-µ-chlorido-[chloridocopper(II)]-di-µ-chlorido-[aquadichloridocopper(II)] chloride Hydrogen-bond geometry (Å, º)

**Table:**

*D*—H···*A*	*D*—H	H···*A*	*D*···*A*	*D*—H···*A*
O1*W*—H1*WA*···Cl2	0.85	2.25	3.097 (4)	172
O1*W*—H1*WB*···O4	0.85	2.38	3.214 (5)	167
O1—H1···Cl6	0.82	2.19	2.995 (4)	169
O3—H3···O2	0.82	1.79	2.522 (5)	147
N3—H3*A*···Cl1	0.82 (5)	2.57 (5)	3.234 (5)	139 (4)
N3—H3*A*···Cl2	0.82 (5)	2.69 (5)	3.292 (4)	132 (5)
C17—H17*A*···O2^ii^	0.97	2.72	3.294 (7)	119

Symmetry code: (ii) −*x*+1/2, *y*−1/2, −*z*.

## References

[bb1] Addison, A. W., Rao, T. N., Reedijk, J., van Rijn, J. & Verschoor, G. C. (1984). *J. Chem. Soc. Dalton Trans.* pp. 1349–1356.

[bb2] Bano, N., Najam, R., Qazi, F., Mateen, A. & Rasheed, A. (2011). *Pak. J. Pharm. Sci.* **24**, 57–63.

[bb3] Bashir, M. & Yousuf, I. (2022). *Inorg. Chim. Acta* **532**, 120757.

[bb4] Dolomanov, O. V., Bourhis, L. J., Gildea, R. J., Howard, J. A. K. & Puschmann, H. (2009). *J. Appl. Cryst.* **42**, 339–341.

[bb5] DrugBank (2023). Levofloxacin (DB01137). Available at: https://go. drugbank.com/drugs/DB01137.

[bb6] Elhusseiny, A. F., El-Dissouky, A., Mautner, F., Tawfik, E. M. & El-Sayed, D. S. (2022). *Inorg. Chim. Acta* **532**, 120748.

[bb7] Freitas, J. T. J., de Melo, C., Viana, O. M. M. S., Ferreira, F. F. & Doruiguetto, A. C. (2018). *Cryst. Growth Des.* **18**, 3558–3568.

[bb8] Freitas, J. T. J., Martins, L. S., Magalhães Calisto, V. F., Beraldo, H. & Diniz, R. (2025). *J. Mol. Struct.* **1345**, 141698.

[bb9] Galani, A., Efthimiadou, E. K., Mitrikas, G., Sanakis, Y., Psycharis, V., Raptopoulou, C., Kordas, G. & Karaliota, A. (2014). *Inorg. Chim. Acta* **423**, 207–218.

[bb10] Golovnev, N. N. & Vasil’ev, A. D. (2016). *Russ. J. Inorg. Chem.* **61**, 1419–1422.

[bb11] Golovnev, N. N., Vasiliev, A. D. & Demina, A. V. (2021). *J. Struct. Chem.* **62**, 236–243.

[bb12] Gorman, E. M., Samas, B. & Munson, E. J. (2012). *J. Pharm. Sci.* **101**, 3319–3330. 10.1002/jps.23200 22610517

[bb13] Groom, C. R., Bruno, I. J., Lightfoot, M. P. & Ward, S. C. (2016). *Acta Cryst.* B**72**, 171–179. 10.1107/S2052520616003954 PMC4822653 27048719

[bb14] Holmes, R. R. (1984). *Prog. Inorg. Chem.* **32**, 119–235.

[bb15] Hooper, D. C. (2001). *Clin. Infect. Dis.* **32**, S9–S15. 10.1086/319370 11249823

[bb16] Huang, P.-S. & Sun, C. C. (2025). *Mol. Pharm.* **22**, 1598–1604. 10.1021/acs.molpharmaceut.4c01307 39913326

[bb17] Kitaoka, H., Wada, C., Moroi, R. & Hakusui, H. (1995). *Chem. Pharm. Bull.* **43**, 649–653.

[bb18] Kumar, M., Kumar, G., Dadure, K. M. & Masram, D. T. (2019). *New J. Chem.* **43**, 15462–15481.

[bb19] Mubarak, A., Abu Ali, H. & Metani, M. M. (2021). *Appl. Organomet. Chem.* **35**, e6428.

[bb20] Nugrahani, I., Sulaiman, M. R., Eda, C., Uekusa, H. & Ibrahim, S. (2023). *Pharmaceutics* **15(1)**, 124. 10.3390/pharmaceutics15010124 PMC9861140 36678753

[bb21] Nugrahani, I., Uekusa, H., Wu, Y., Hori, T., Auliaurridho, H., Herawati, D., Panjaitan, F. E., Garmana, A. & Wibowo, M. S. (2025). *J. Pharm. Sci.* **114**, 103779. 10.1016/j.xphs.2025.103779 40187736

[bb22] Owens, R. C. & Ambrose, P. G. (2000). *Clin. Infect. Dis.* **31**, 118–125.

[bb23] Parsons, S., Flack, H. D. & Wagner, T. (2013). *Acta Cryst.* B**69**, 249–259. 10.1107/S2052519213010014 PMC3661305 23719469

[bb24] Podder, V., Patel, P. & Sadiq, N. M. (2024). *StatPearls [Internet].* Treasure Island, FL: StatPearls Publishing.

[bb25] Rigaku OD (2022). *CrysAlis PRO*. Rigaku Oxford Diffraction Ltd, Yarnton, Oxfordshire, England.

[bb26] Scholar, E. M. & Pratt, W. B. (2000). In *The antimicrobial drugs*, 2nd ed. Oxford University Press.

[bb27] Sheldrick, G. M. (2015*a*). *Acta Cryst.* A**71**, 3–8.

[bb28] Sheldrick, G. M. (2015*b*). *Acta Cryst.* C**71**, 3–8.

[bb29] Singh, S. S. & Thakur, T. S. (2014). *CrystEngComm* **16**, 4215–4224.

[bb30] Ueda, H., Tse, J. Y., Miyano, T., Nakayama, Y., Mo, P., Hatanaka, Y., Uchiyama, H., Tozuka, Y. & Kadota, K. (2025). *RSC Pharm.* **2**, 264–278.

[bb31] Vasiliev, A. D. & Golovnev, N. N. (2019). *J. Struct. Chem.* **60**, 1959–1964.

[bb32] Vasiliev, D. & Golovnev, N. N. (2011). *J. Struct. Chem.* **52**, 930–934.

[bb33] Wang, X.-S., Tang, Y.-Z. & Xiong, R.-G. (2005). *Chin. J. Inorg. Chem.* **21**, 1275.

[bb34] Westrip, S. P. (2010). *J. Appl. Cryst.* **43**, 920–925.

